# Automatic Lane Segmentation in TLC Images Using the Continuous Wavelet Transform

**DOI:** 10.1155/2013/218415

**Published:** 2013-09-19

**Authors:** Bruno Moreira, António Sousa, Ana Maria Mendonça, Aurélio Campilho

**Affiliations:** ^1^INEB-Instituto de Engenharia Biomédica, Campus da FEUP, Universidade do Porto, Rua Dr. Roberto Frias, s/n, 4200-465 Porto, Portugal; ^2^Faculdade de Engenharia da Universidade do Porto (FEUP), 4200-465 Porto, Portugal; ^3^Instituto Superior de Engenharia do Porto (ISEP), Instituto Politécnico do Porto, 4200-072 Porto, Portugal

## Abstract

This paper describes a new methodology for lane detection in Thin-Layer Chromatography images. An approach based on the continuous wavelet transform is used to enhance the relevant lane information contained in the intensity profile obtained from image data projection. Lane detection proceeds in three phases: the first obtains a set of candidate lanes, which are validated or removed in the second phase; in the third phase, lane limits are calculated, and subtle lanes are recovered. The superior performance of the new solution was confirmed by a comparison with three other methodologies previously described in the literature.

## 1. Introduction

This paper focuses on one of the initial components of a screening tool for Fabry disease (FD), based on the automatic analysis of Thin-Layer Chromatography (TLC) images: the lane segmentation. FD is a rare X-linked hereditary metabolic storage disorder caused by genetic abnormalities, which leads to an enzymatic deficiency [1] resulting in the accumulation of excessive quantities of one class of lipids, the sphingolipids, globotriaosylceramide (Gb3) being the most prevalent in FD patients [2]. Although the usual onset of the first symptoms is in childhood, by middle-age, life-threatening complications are often developed in untreated patients [3]. The recent availability of enzymatic replacement therapy, in conjunction with the progressive nature of the disease, has renewed the interest in this disorder and revealed the need for early diagnosis, which can only be achieved with generalized screening programs [4].

The complete diagnosis of FD is very complex, but the first phase is simply based on the detection of an abnormal quantity of Gb3 in urine or blood plasma of the patient. The direct measurement of these compounds can be carried out by using a microtandem mass spectrometer (MS/MS), but its use is very expensive. Another approach, less expensive, is the analysis of patient urine or blood plasma samples using TLC [5]. TLC is a type of liquid chromatography that allows the separation, identification, and visual quantification of a wide variety of components in a mixture [6]. The components to be separated by the chromatographic process are distributed between two phases, a stationary phase and a mobile phase. The solutes, distributed preferentially in the mobile phase, will move more rapidly through the system than those distributed preferentially in the stationary phase. Thus, the solutes will elute in order of their increasing distribution with respect to the stationary phase [7]. At the end of the chromatographic process, the components are spread along a lane and distributed by different bands based on their physical properties (size, molecular weight, etc.) [6]. TLC has the highest sample throughput amongst the chromatographic techniques. Up to 30 different samples and standards can be applied to a single plate in individual lanes and be analyzed at the same time, which explains the spreading use of TLC, as both a screening and confirmation tool, worldwide [8]. 

For the implementation of a screening tool for Fabry disease, the identification of normal and suspicious individuals will be based on the presence or absence of the disease biomarkers, previously separated by the TLC development of urine samples. The screening system is supported by a set of image analysis and classification methods, so as to try to automate the interpretation of TLC chromatograms. One initial and crucial phase, usually called lane segmentation, involves the automatic separation of the individual samples contained in the digital image of a TLC plate.

Several software packages that include solutions for lane segmentation can be found in the literature, although most of them were developed for gel electrophoresis images. KODAK 1D [9] software has an “automatic lane finder” option that uses a multiple pass algorithm to determine the lanes on the image, as an alternative to the interaction with the operator. GelBuddy [10] is a user-friendly Java-based software that requires the number of lanes as input. The lane tracks are located by detecting local maxima of intensity profiles, obtained through the integration of pixel values over a set of horizontal sectors. Getlanes [11] operates on four-color, fluorescence-based, and electrophoretic gel images. Each gel file contains four filter images, which are summed to form a brightness image. The “brightness” profile is obtained by a vertical integration of the brightness image, and a first-difference approximation to the gradient is computed to identify maxima, which are marked as lane locations. It also uses models of expected lane and interlane spacing and lateral lane behavior to improve tracking on imperfect gels. PyElph [12] is a software tool for gel image analysis and phylogenetics. For lane detection, it computes the maximum value of each column and creates an intensity profile. A threshold set to 70% of the maximum intensity value of the domain is used for lane detection. The lanes selected at this threshold level are used for calculating the mean value for the lanes' width. The lanes narrower than the mean width are removed from the set, and the mean width of the remaining lanes is again computed. Finally, all the lanes between two thresholds (70% and 15% of the maximum of the domain), and presenting a width deviation of less than 25% from the mean width, are included in the final set. LaneRuler [13] approaches the problem by dividing the data region into “zones” such that each zone has the same integrated intensity, thus placing more “nodes” in data-rich portions of the gel compared to its data-poor portions. Generic lane width is then determined based on a Fourier analysis in each zone.

Methods developed for lane segmentation include algorithms supported by the application of spatial-domain filters [14] and semiautomatic detection based on the assumption that the lanes have constant width and are equispaced [15]. Bajla et al. [16] proposed a semiautomatic method for lane separation in electrophoretic gel images. This approach is based on a one-dimensional cumulative indicator of lane edges, coupled with a shifted regular initial grid calculated from the *a priori* information on the number of lanes. In [17], the bands contained in each lane are enhanced and afterwards replaced by their skeletons. Then, lane segmentation is performed based on these band skeletons. In [18], two methods are proposed, one based on an iterative moving average filter (IMA) and the other using the continuous wavelet transform (CWT). 

Sousa et al. [19] presented an automatic procedure for lane detection in TLC images based on the detection of local extreme points in the image projection profile, which had been previously smoothed using a nonweighted moving average filter. In the approach described in [20, 21], lane detection is accomplished by locating the lane boundaries. The derivative of image intensities in the horizontal direction is calculated, and its values are summed across the vertical direction. The resulting one-dimensional curve has local extremes at the boundaries of the lanes. In [22], the image is horizontally divided into equal parts, and for each one a profile is obtained from its vertical projection. Each profile is smoothed and used to estimate the lane centers (profile maxima) in each image part. The methodology connects local maxima along a lane, by visiting each partitioned image from the bottom to the top, and checking whether two local maxima are within a range of horizontal coordinates. Other methods related to lane detection include algorithms to geometrically correct images with the help of distance maps [23] and active shape models [24].

Most of the packages and methods that were developed for gel image analysis assume some regularity in lane distribution or require additional inputs, such as the number of lanes in the image. So, this kind of solution does not work well when the images do not present the expected regularity in lane distribution, as occurs in some images of our dataset. Therefore, an approach that is able to deal with the specific characteristics of TLC images, without lowering performance when applied to more standard ones, is required in our system. 

This paper presents a new methodology for automating the detection of lanes in digital images of TLC plates, which is an improved version of the algorithm presented in [25]. The main difference between the two methods is the initial smoothing step, which is applied to the intensity profile resulting from the integration of the image area containing the lanes. The initial smoothing step is essential for the success of the following lane segmentation steps, as it allows the removal of noise and irrelevant data. In [25], a Savitzky-Golay (SG) filter [26] was applied to the intensity profile, while in the solution herein proposed, the Continuous Wavelet Transform (CWT) [27] is used for removing both noise and other high frequency components. When compared to other algorithms in the literature, our approach also relies on the detection of extreme points of the intensity profile that results from the projection of the image data. However, both in the method herein described and in [25], after locating the most obvious lanes associated with the extreme points of the smoothed profile, two further steps are performed, one for validating previously included lanes and removing false detections and another for adding very subtle lanes that could not be clearly distinguished. Nevertheless, a new implementation of these two supplementary steps is now proposed using image adaptive parameters instead of the constant settings used in [25].

The outline of the paper is as follows.  Section 2 describes the proposed method, including the preprocessing sequence applied to the images, the CWT-based technique for smoothing the intensity profile, and the lane segmentation process. The dataset description, lane segmentation examples, and some performance measures are presented in Section 3. Furthermore, the proposed methodology is compared with three previous solutions, described in [18, 19, 25]. Results are discussed in Section 4. Some conclusions and further research are included in Section 5.

## 2. Methodology for Lane Segmentation

### 2.1. Overview of the Methodology

**Figure 1:**
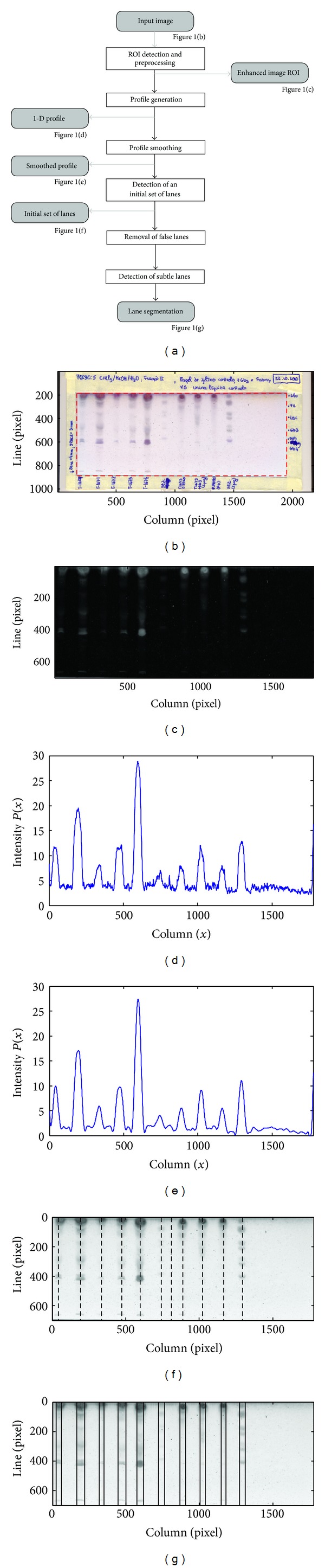
Main steps of the methodology: (a) block diagram; (b) original TLC image; (c) enhanced image ROI; (d) ROI intensity profile; (e) smoothed profile; (f) initial set of lanes; (g) final lane segmentation.

The processing flow of the method for lane segmentation herein proposed is illustrated in the block diagram of Figure 1(a). The original RGB image is acquired, and the region of interest (ROI) is obtained, as presented by the dashed rectangle in Figure 1(b). The ROI is preprocessed, and the enhanced image is shown in Figure 1(c). The resulting image columns are integrated in order to obtain an intensity profile, which is then smoothed using the technique described in detail in Section 2.3. Figures 1(d) and 1(e) show the initial and smoothed profiles, respectively, for the image data of Figure 1(c). The smoothed profile will allow the selection of an initial set of potential lanes such as those in Figure 1(f), which is afterwards updated with the removal of false detections. The last phase focuses on the detection of missed lanes and on the localization of lane centers and boundaries, as can be observed in Figure 1(g).

### 2.2. Image Acquisition and Preprocessing

At the end of the chromatographic process, TLC plates quickly deteriorate, and therefore they need to be scanned as soon as possible. The TLC digital image is acquired in true color, since the specialist will need this information for inspecting the images later, as it helps in the identification of specific biomarkers. A typical TLC image, such as the one shown in Figure 1(b), usually contains two distinct regions: a border and a region of interest (ROI). The border is the external region of the image, and it comes from the adhesive tape used for protecting the plate right after the chromatographic process. This adhesive tape usually has handwritten text with the identification/composition of the samples. It has no relevant information for the image analysis process, but it interferes with the correct detection of the lanes, so this region of the image is discarded. The ROI is the internal region of the image, formed by lanes containing the result of chromatographic development and empty spaces. This is the relevant region for our methodology, which is automatically delineated using a classification-based algorithm described in [28].

The ROI is converted from RGB to grayscale (GS) by retaining the luminance information. The resulting image (GS ROI) is represented using 256 levels of gray and is computed by a weighted sum of the red (R), green (G), and blue (B) components given by

(1) $$\begin{array}{c} \text{GS ROI}=0.30\text{R}+0.59\text{G}+0.11\text{B}. \end{array}$$

The GS ROI is then processed in order to remove the background, which is estimated using a closing morphological operator with a square structuring element [29]. The size of the morphological operator is derived from the size of the image and is equal to 10% of the number of image rows, thus ensuring that the structuring element length is higher than the size of the bands. The estimated background is subtracted from the GS ROI, and the image data is projected onto the direction perpendicular to lane development (vertical projection) to integrate the information into an intensity profile that will be used for lane detection. The intensity profile *P*(*x*) of an image *I*(*x*, *y*) is obtained by averaging the grey levels on each column in the image as defined by

(2) $$\begin{array}{c} P\left(x\right)=\frac{1}{N}\sum_{y=1}^{N}I\left(x,y\right),\;x=1,\ldots ,M, \end{array}$$

where *M* is the number of columns and *N* is the number of rows in the image.

Since during background removal the image is inverted (as can be observed in Figure 1(c)), the intensity profile presents maximal regions where the original image is darker (lane zones) and minimal regions where this image is lighter (empty zones). It is worth mentioning that TLC images are often corrupted by noise on the top rows due to a significant accumulation of compounds that are present in biological materials. This noise makes the image analysis process more difficult, as it influences the profile, and smoothes the transition between regions with and without lanes. Moreover, the top region of a TLC image is not important for the remaining phases of the method, as it does not contain any relevant compounds used as biomarkers. To overcome this problem, we decided to exclude the top 25% of rows from the averaging into the intensity profile. 

### 2.3. Profile Smoothing

The intensity profile obtained by the previously mentioned projection usually presents local variations that can lead to a high number of false lanes. Thus, a smoothed version of the profile, containing just the main intensity variations corresponding to the transitions between lanes zones and empty zones, is required. Moving average filters have been the most common solution applied in other methods for lane segmentation, but this kind of smoothing filter can destroy important signal information. For instance, the peaks of the intensity profile corresponding to the center of the lanes lose height when submitted to a moving average filter. The ideal filter would produce smoothed data without flattening the peaks. To overcome these problems, we propose a smoothing approach based on the CWT.

The Wavelet transform offers simultaneous interpretation of the signal in both time and frequency, which allows local, transient, or intermittent components to be elucidated [30]. The wavelet transform provides a series expansion of a signal, using a set of orthonormal-based functions, which are generated by scaling and translation of two functions: the mother wavelet and the scaling function (daughter wavelet). As a result of wavelet analysis, a family of hierarchically organized decompositions is produced, where each level of the hierarchy is associated with a specific scale [27].

Although the discrete wavelet transform (DWT) is a common choice in many applications, we selected the CWT for this particular application because the highest scale resolution is provided by the continuous transform. In the DWT, scales are chosen so that the wavelets are orthogonal, which implies that the scale range will be the smallest one that will not produce loss of information [31]. The scales in the CWT are not constrained, and the wavelets are nonorthogonal. This property, while making the CWT redundant, provides a finely detailed description of a signal in terms of both time and frequency [32]. These characteristics allow an accurate selection of scales that will be important in the smoothing of the intensity profile. 

The CWT uses a set of wavelets, where each element is constructed from the same function, the original wavelet *ψ*(*t*) (mother wavelet). Each daughter wavelet is a scaled and shifted version of the mother wavelet [33], according to

(3) $$\begin{array}{c} \psi_{\left(s,\tau \right)}\left(x\right)=\frac{1}{\sqrt{s}}\psi \left(\frac{x-\tau }{s}\right), \end{array}$$

where *s* and *τ* are the scale and translation parameters, respectively [33]. The daughter wavelets include an energy normalization term, $1/\sqrt{s}$, that keeps the energy of these wavelets equal to the energy of the original mother wavelet. For this application, the Morlet wavelet was used as the mother wavelet function, because it is known for its excellent time-frequency localization [34]. It consists of a plane wave modulated by a Gaussian function and is defined by

(4) $$\begin{array}{c} \psi_{0}\left(\eta \right)=\pi^{-1/4}e^{i\omega_{0}\eta }e^{-\eta^{2}/2}, \end{array}$$

where *ω*_0_ is the nondimensional frequency and *η* is a nondimensional “time” parameter. To satisfy the wavelet's admissibility condition, this function must have a zero mean and be localized in both time and frequency spaces [35].

In our application, for the analysis of the profile resulting from the projection of the ROI data, the scales of interest in the CWT are related to the width of profile peaks, being the most significant ones determined by the presence of lanes. Hence, the most interesting coefficients for reconstructing a reliable smoothed version of the original profile are those whose scales correspond to the expected range of lane widths. After the analysis of the CWT decomposition of several intensity profiles, it was possible to identify a common pattern consisting of three ranges in the scale domain. At low scales, several significant coefficients are generated by high frequency noise (noise range). On the other hand, at very high scales, only the coefficients associated with the profile baseline are found (baseline range). So, the coefficients that will contain lane information are normally located in the middle range and usually present the highest amplitude values (lane range). 

In order to enhance lane information and at the same time achieve an adequate smoothing, the intensity profile should be reconstructed using only the scales belonging to the lane range, defined by the two cut-off values for scales: the first, herein called *cutoff-min*, should separate the lane range from the noise range, while the second one, *cutoff-max*, sets apart the lane and baseline ranges. Both cut-off values should be situated within the lane range, which is limited by scales 30 and 250.

**Figure 2:**
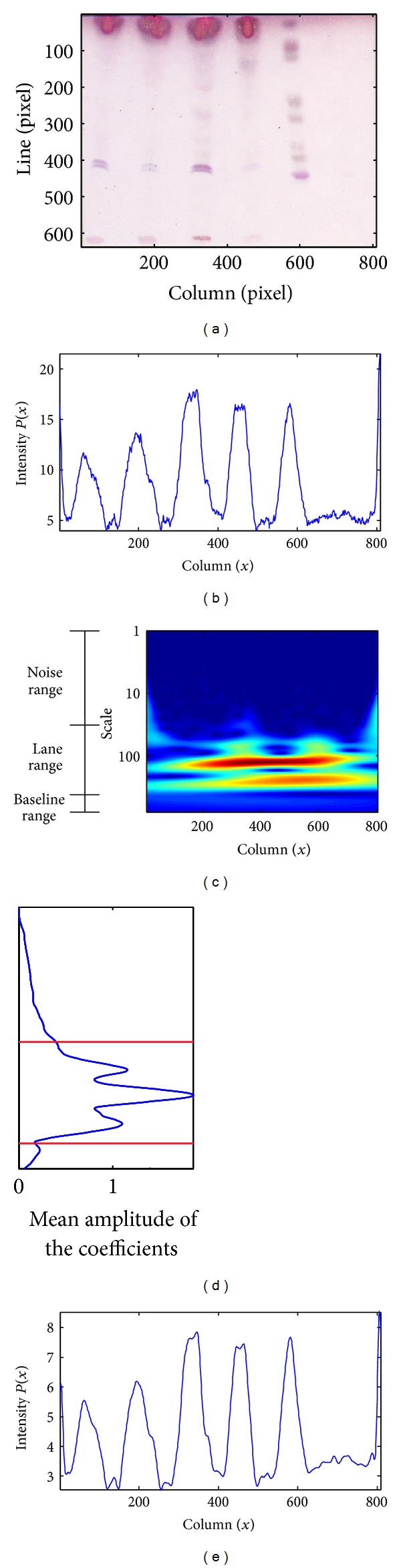
(a) ROI of a TLC image. (b) Profile intensity obtained for the same image. (c) Color map representation of the CWT coefficients for the intensity profile. (d) The lane range limits are represented by the red lines. (e) Smoothed profile resulting from the reconstruction of the scales within the lane range.

Figure 2 shows an overview of the smoothing process using the CWT.  Figure 2(a) presents the ROI of a TLC image, whose integration into a 1D profile leads to the result depicted in Figure 2(b). The CWT coefficients for this intensity profile are presented in Figure 2(c), where each row represents one scale, with the higher scales standing at the bottom. The amplitude of each coefficient is encoded using a color map, ranging from dark blue for low values to red for high values. The noise, lane and baseline ranges can be easily identified. In Figure 2(d), the signal containing the mean amplitude of the coefficients for each scale (in the same row) is shown. The coefficients in the predefined lane range were used for the reconstruction of the smoothed profile depicted in Figure 2(e).

For each particular image, adaptive cut-off values are chosen within the predefined lane range. The selected value for the *cutoff-min* threshold represents a tradeoff between noise reduction and the detection of thin lanes. A *cutoff-min* at a higher scale, thus excluding more high frequency coefficients, would ensure a better smoothing than at a lower scale, but the information of small width lanes may be excluded from the reconstructed signal. The choice of *cutoff-max* should take into account that a high value may prevent the detection of subtle lanes, by hiding them with the inclusion of very low frequency components (baseline information). However, a low *cutoff-max* will lead to false lane detections if the image has a large, empty zone, without lanes. 

Based on the analysis of the signal containing the mean amplitude of the coefficients for each scale, we concluded that the highest peak in the predefined lane range contains almost all lane information. However, higher scales may also contain relevant information for avoiding false detections in empty zones of the profile. Thus, to find the adequate cut-off values for each profile, the signal containing the mean amplitude of the coefficients for each scale is searched for its maximum in the scale interval that corresponds to the predefined lane range. The *cutoff-min* is set to the scale value which corresponds to the local minimum of the signal immediately before this maximum. If there is another local maximum at a higher scale, the *cutoff-max* value is the scale of the local minimum immediately after this local maximum; otherwise, the separation between the lane and background regions is not as straightforward, and the *cutoff-max* value is set at the highest scale in order to prevent loss of important information (i.e., all the higher scales will be included).

**Figure 3:**
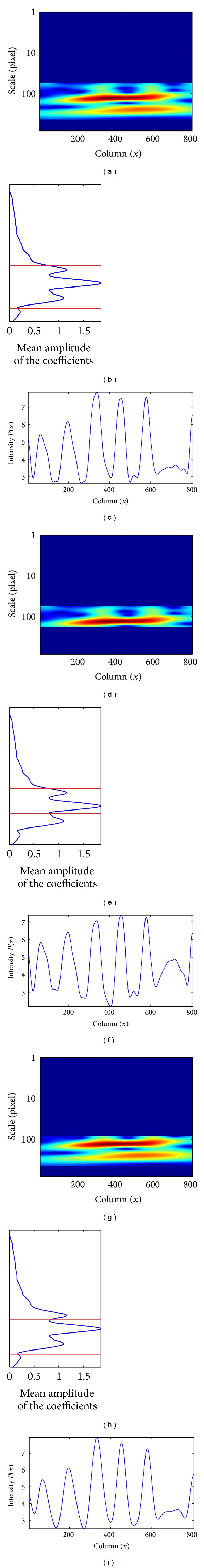
(a) Representation of the scales used to smooth the intensity profile. (b) *Cutoff-min* set to 50. (c) The effect of the exclusion of lower scales is reflected in the reconstructed profile. (d) Representation of the scales used to smooth the intensity profile. (e) *Cutoff-max* set to 150. (f) The exclusion of scales containing low frequency information may lead to the appearance of false lanes. (g) Representation of the scales used to smooth the intensity profile. (h) Cutoff values chosen using the proposed methodology (scales 80 and 250). (i) Reconstructed profile.

Three examples of how different cut-off ranges affect the reconstruction of the profile are presented in Figure 3, for the TLC image in Figure 2(a). The images in the first row of Figure 3 illustrate the influence of the *cutoff-min*, which was set to scale 50. The result, shown in Figure 3(c), is better than the one obtained in Figure 2(e). In the second row, the *cutoff-max* value was reduced to scale 150. The range of scales used for the profile reconstruction is shown in Figure 3(d) and Figure 3(e). Due to the exclusion of important scales, the resulting profile, depicted in Figure 3(f), now contains a local maximum in an empty zone of the image, which could lead to a false detection. Finally, in Figures 3(g)–3(i), there are the results obtained using the automatic process used for selecting the two cut-off values. 

### 2.4. Lane Segmentation

#### 2.4.1. Detection of an Initial Set of Lanes

Lane segmentation is performed using the smoothed profile that results from the reconstruction using CWT coefficients in the scale range (*cutoff-min, cutoff-max*). As a general rule, the local maxima regions of the profile should correspond to occupied lanes, and the local minima regions should correspond to the space between them. These regional extremes are searched for on the results of two morphological transformations, *h*-maxima and *h*-minima. These transformations suppress all the extremes whose heights are less than a given threshold *h*. For the *h*-maxima transformation, this is achieved by performing the reconstruction *R*_*f*_^*δ*^ by dilation of *f* from *f* − *h* [36]

(5) $$\begin{array}{c} \text{HMA}{\text{X}}_{h}\left(f\right)=R_{f}^{\delta }\left(f-h\right). \end{array}$$

By analogy, the *h*-minima transformation is defined as

(6) $$\begin{array}{c} \text{HMI}{\text{N}}_{h}\left(f\right)=R_{f}^{\delta }\left(f+h\right). \end{array}$$

The results of the detection of extremes are two binary functions, one marking the regions of the profile which are local maxima and the other marking the local minima. These two binary functions are combined into a single one, which is true for regions corresponding to local maxima but not marked as local minima. The integration of information regarding local minima is important, to prevent the inclusion of regional maxima of very low intensity. Each region of this combined profile is considered as a potential lane, whose center and width are, respectively, the middle point and the width of the region. 

**Figure 4:**
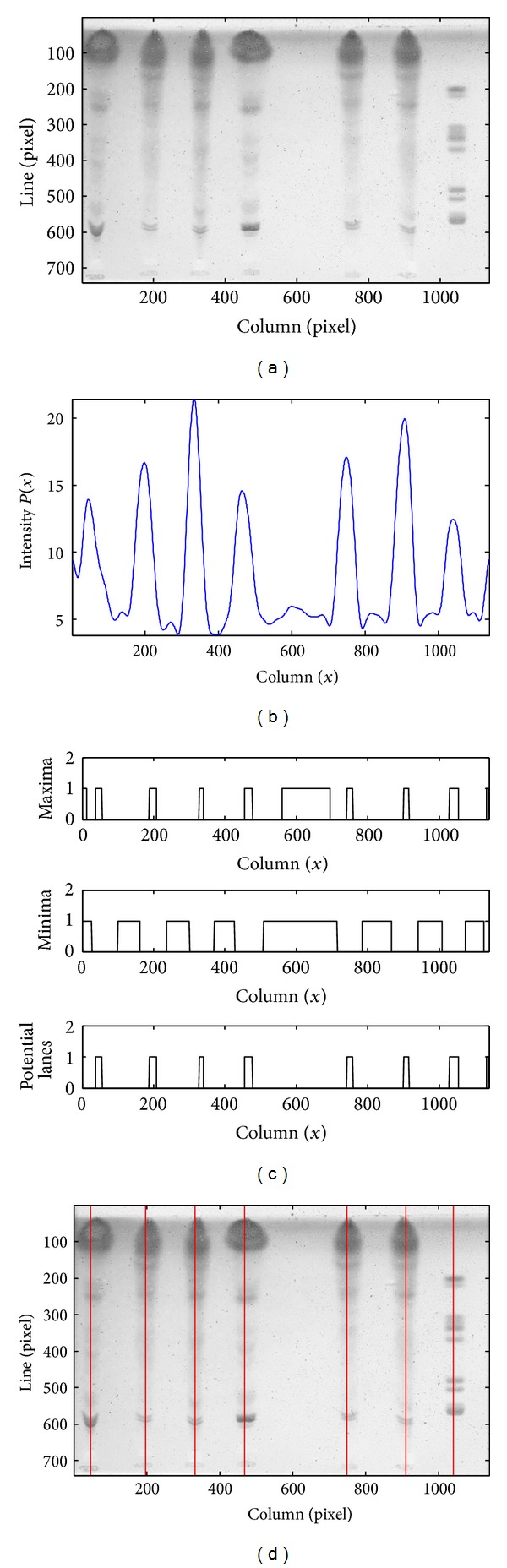
(a) ROI of a TLC image. (b) Smoothed intensity profile. (c) Regional maxima (top), regional minima (middle), and set of potential lanes resulting from their combination (bottom). (d) Initial set of lanes represented by their central lines.

The selection of an initial set of lanes is exemplified in Figure 4. The ROI of a TLC image after the preprocessing step is presented in Figure 4(a), and Figure 4(b) depicts the smoothed profile obtained with the CWT technique.  Figure 4(c) shows the two binary functions with regional extremes together with their combination, which defines the initial set of lanes, whose central positions are represented by the vertical lines in Figure 4(d).

#### 2.4.2. Removal of False Lanes

The validation of the initial set of lanes is based on three measures: relative lane width, lane distance, and lane intensity. An initial estimation of lane widths is obtained from the signal resulting from the combination of the detected regional extremes. For each identified lane, the maximum value that occurs within its region is defined as the lane intensity. The distance between the central positions of two consecutive lanes (lane distance) is also measured. For each image, the mean and standard deviation of lane width (*m*_*w*_, std_*w*_), lane distance (*m*_*d*_, std_*d*_), and lane intensity (*m*_*i*_, std_*i*_) are also calculated.

All the lanes that have intensity below half of the mean lane intensity, *m*_*i*_/2, will be removed if either the width is outside the range defined by *m*_*w*_ ± std_*w*_ or if the distance to one of the adjacent lanes (or image border, for the lanes in the image extremes) is below a threshold value established by *m*_*d*_ − std_*d*_.

**Figure 5:**
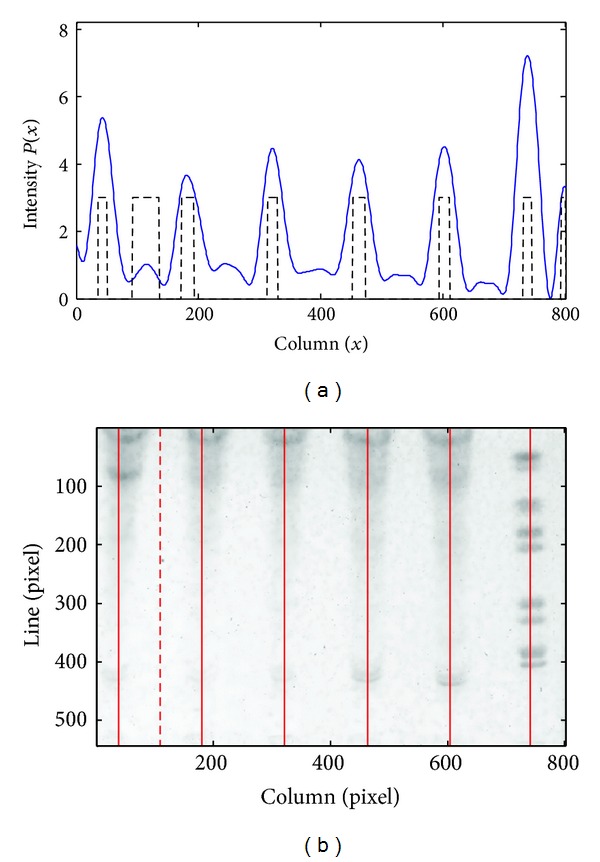
(a) Intensity profile (continuous line) and results of the first phase of lane detection (initial set represented by the dashed line); (b) the dashed line represents the removed lane.

The removal of a false lane is shown in Figure 5. The intensity profile obtained after smoothing is depicted in Figure 5(a) by the solid line. The dashed line represents the outcome of the initial set of lanes. In Figure 5(b), a false detection, represented by the dashed line, was removed at this stage, due to the proximity to the adjacent lanes.

#### 2.4.3. Recovery of Missed Lanes and Detection of Lane Limits

This step is mainly based on the analysis of the derivative of the profile. For each true lane, there are two local extremes in the derivative of the profile, a local maximum and a local minimum, that can be associated with lane limits. These extreme points allow accurate lane segmentation, since they are related with the inflection points of the intensity profile usually delimitating the transition between lanes and empty zones. Because of the intensity fluctuations that are present in the derivative, the two local extreme points that are considered are those with the highest and lowest amplitude values occurring on opposite sides of the lane. When all the lane limits are determined, we can get the mean lane width, *mlw*, along with the mean difference between the amplitude values of two corresponding extreme points in the profile derivative, *mla*. Both values are used as references to look for low intensity lanes.

After detecting the limits for all validated lanes, some “empty zones” (zones without detected lanes) can still remain in the profile. For those regions which have width larger than the *mlw*, the local extremes of the derivative are retrieved and grouped into maximum-minimum pairs. For each pair, the distances between the extreme positions and their amplitude difference are compared with the two previously mentioned reference values, in order to decide on the inclusion of this potential lane into the set of validated lanes. For each “empty zone,” more than one lane can be recovered using Algorithm 1.

**Figure 6:**
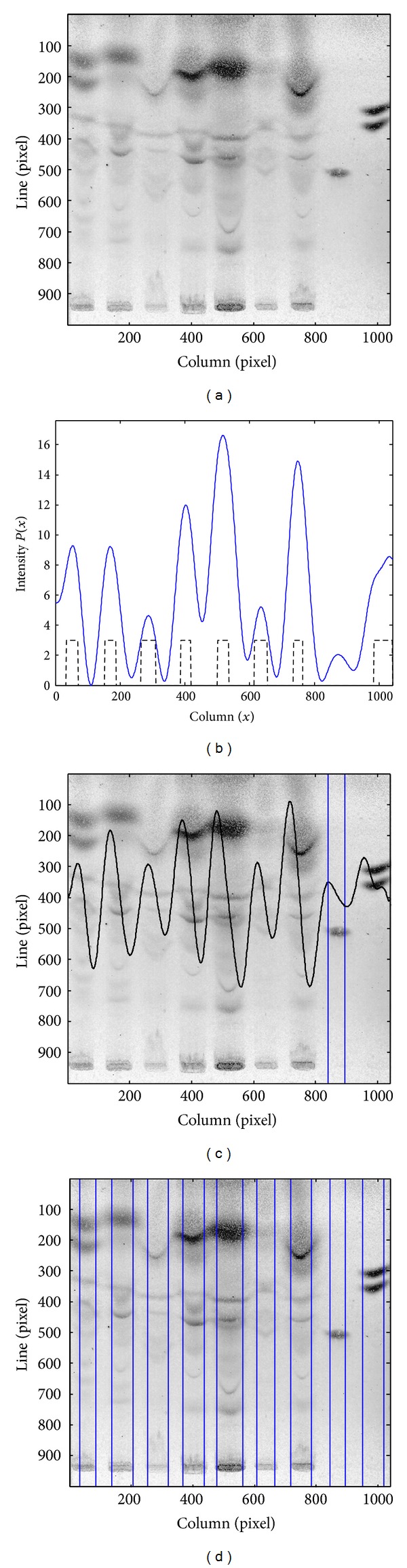
(a) ROI of a TLC image; (b) Intensity profile (continuous line) and results of the first phase of lane detection (initial set represented by the dashed line); (c) Image ROI with its profile derivative overlapped; (d) Results after the three phases of the lane detection process, with the boundaries of the detected lanes represented by the vertical lines.

Figure 6 presents an example of the lane recovery step, as well as the final lane segmentation result. The smoothed intensity profile for the image presented in Figure 6(a) is depicted in Figure 6(b), and one lane (the 8th) was not included in the initial set due to its low intensity.  Figure 6(c) shows the ROI of the image with the profile derivative overlapped. The analysis of the profile derivative allows the detection of the missed lane, thus leading to the updated set in Figure 6(d) with the lanes properly segmented. 

## 3. Results

### 3.1. Datasets

The proposed methodology was evaluated using two distinct datasets. These two datasets have in common the presence of empty lanes as a result of the sample acquisition protocol. Immediately before the start of the TLC analysis process, a set of tasks is sequentially applied to each sample in order to increase the concentration of biomarkers. However, when the samples are applied to the plate, some of them may not be prepared for analysis and thus will not run correctly in the mobile phase, leading to the formation of empty lanes in between occupied lanes. Furthermore, a shortage of samples causing incomplete filling of a TLC plate may lead to a region of empty lanes. These situations happen more often in the first dataset (DB1), which contains 66 images (651 lanes), the majority of which were obtained using human urine and blood. The images of this dataset are also characterized by a high variability in size, resolution, and number of lanes. The second dataset (DB2) is composed of 169 images (1422 lanes) of TLC plates, containing urine chromatograms of individuals suffering from several lysosomal storage diseases [37]. 

**Figure 7:**
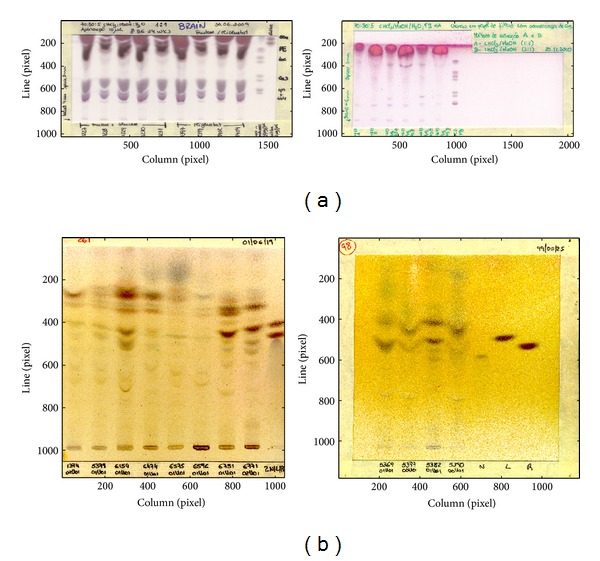
(a) Images from DB1. (b) Images from DB2.

Due to size differences, all the images were rescaled to a fixed number of lines (1024), while keeping the columns-lines ratio constant (rescaling factor = 1024/initial number of lines). A bicubic interpolation function was used to calculate the intensities of the rescaled image. As a result of this resizing operation, the sizes of lanes and bands become more or less identical, independent of the original image size.  Figure 7 contains four TLC images demonstrating the differences between the two datasets.

### 3.2. Parameter Settings

The proposed methodology is supported by a set of values that can be separated into two distinct classes: reference values and constant parameters. Reference values are adaptive quantities, automatically calculated for each specific image and can vary from image to image. Parameters are preestablished numerical values and are identical for each image of the dataset.

The algorithm depends on three parameters that should be specified for each dataset: the *h* value, the *ω*_0_ value of the Morlet wavelet, and the scale range where the *cutoff-min* and *cutoff-max* values are searched for. The *h* value used in the *h*-maxima and *h*-minima transformations was established as a percentage of the image intensity profile maximum; this percentage value was set as 5% for DB1 and 10% for DB2. The parameter *ω*_0_ of the Morlet wavelet was set to 6 to satisfy the admissibility condition. A specific scale range (ranging from scales 30 to 250 in both datasets) was used for searching the *cutoff-min* and *cutoff-max* values. These two limits were established based on the spatial distance between lanes.

The reference values used in the false lane removal and subtle lane recovery phases are adaptive quantities that are calculated for each specific image. The false lane removal uses the mean value and standard deviation of the lane's width, distance, and intensity, as criteria, to validate the initial set of lanes. In the lane recovery phase, a new lane is accepted if its width is larger than 60% of *mlw* and the difference between its extreme values is larger than 30% of *mla*. These values were fixed after the observation of a set of empty lanes. 

**Figure 8:**
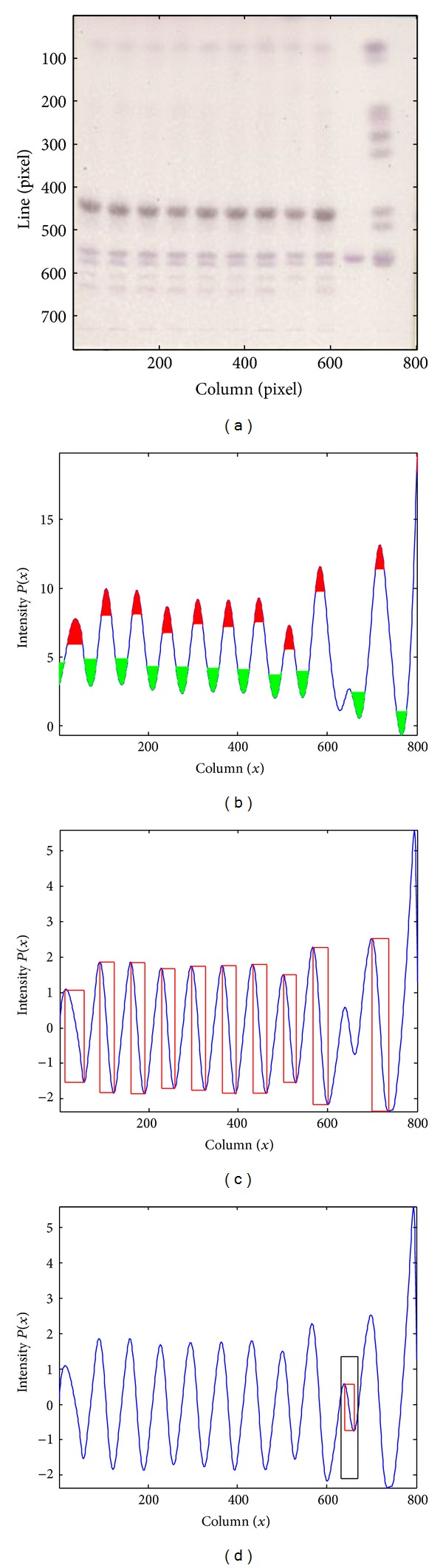
(a) ROI of a TLC image. (b) Intensity profile with the representation of the regions selected by the *h*-maxima and *h*-minima transformations. (c) Profile derivative. The width and amplitude of each lane are represented by the width and height of each rectangle, respectively. (d) The mean values for the lane width and amplitude are represented by the outside rectangle, while the values obtained after the search for subtle lanes are represented by the inner rectangle.

The lane recovery process is represented in Figure 8. The maxima and minima regions selected by the *h*-transformations for the profile of the original image of Figure 8(a) are shown in Figure 8(b) in red and green, respectively. In Figure 8(c), the width and amplitude of each lane in the initial set are represented by a rectangle whose sides are equal to those lane features.  Figure 8(d) shows a lane recovered in the last phase of the algorithm, where the outside rectangle has dimensions *mlw* and *mla*; the mean values obtained after averaging for the lanes present in Figure 8(c), and the inside rectangle represents the corresponding values for the recovered lane.

### 3.3. Comparison of Lane Segmentation Methods

In this subsection, some results of application of the proposed methodology are presented and compared with those obtained using the methods described in [18, 19, 25].

**Figure 9:**
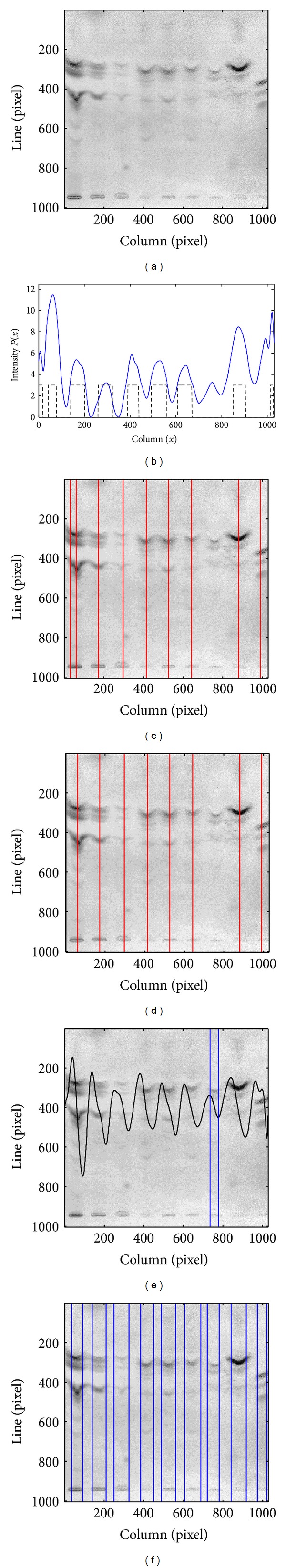
Application of the proposed methodology to a DB2 image. (a) ROI of the original TLC image. (b) Smoothed intensity profile. (c) Initial set of lanes represented by the vertical lines. (d) Set of lanes after the false lane removal phase. (e) Profile derivative overlapped on the image, with the limits of the recovered lane represented by the vertical lines. (f) Final result of lane segmentation.

Figure 9 illustrates the different phases of the herein proposed method for a DB2 image, whose ROI is shown in Figure 9(a). The intensity profile of Figure 9(b) was obtained after smoothing using the CWT. The regions marked by the dashed line are the result of the first phase of lane detection and include seven true lanes correctly located, one true lane incorrectly detected because two central positions (one correct and one false) were assigned for that single lane, and one true lane missed. These results can be observed in Figure 9(c) where the vertical lines correspond to the center of the lanes detected by the algorithm. The false central line was removed in the second phase, and the updated set of lanes is shown in Figure 9(d). The third phase allows the recovery of the missed lane (Figure 9(e)), along with the determination of lane boundaries as depicted in Figure 9(f).

**Figure 10:**
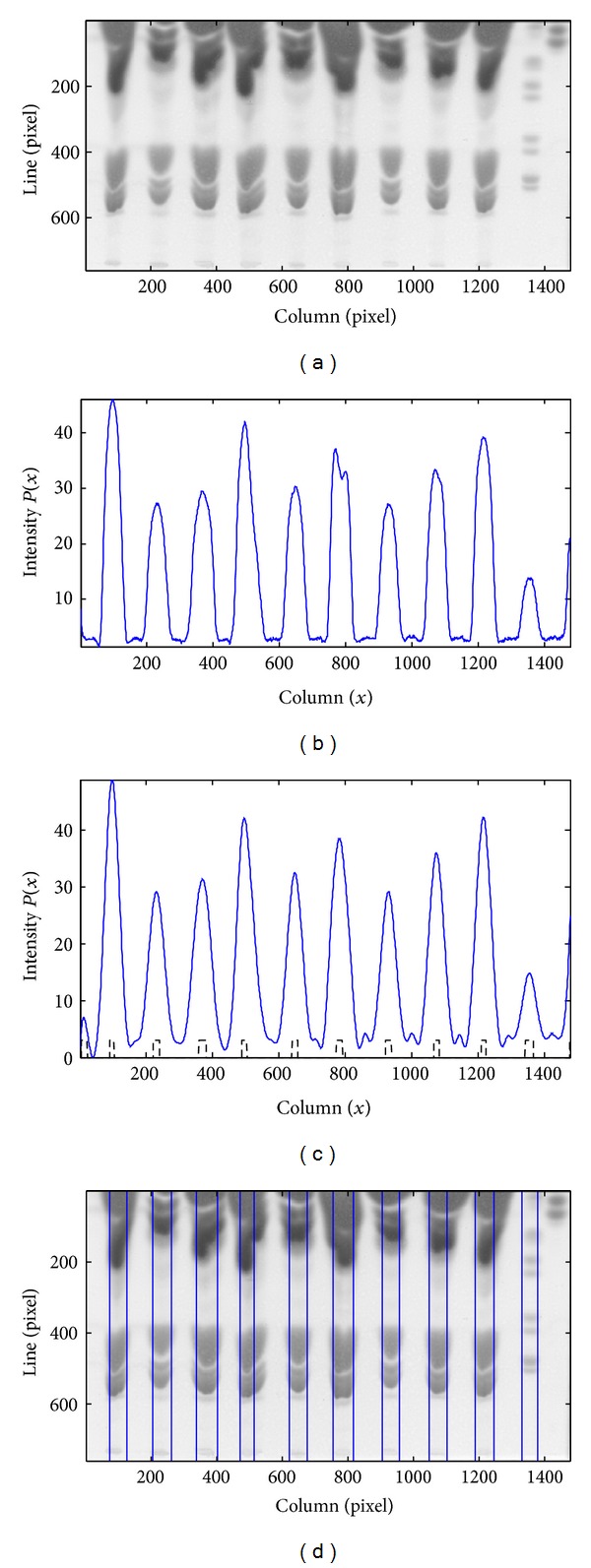
(a) ROI of the original TLC image. (b) Original intensity profile. (c) Smoothed intensity profile obtained with the CWT approach and initial set of lanes represented by the dashed lines. (d) Boundaries of the final set of lanes.

The image shown in Figure 10(a), selected from DB1, presents an example where the methodology fails to correctly detect all the existent true lanes. At the final phase, there is still one undetected lane, on the right of the image. This lane has a band located on the top rows that are not included in the intensity profile. However, it is worth mentioning that lanes presenting bands only in the image top rows are not important for our specific application, because FD biomarkers are located in the bottom half of the image.

**Figure 11:**
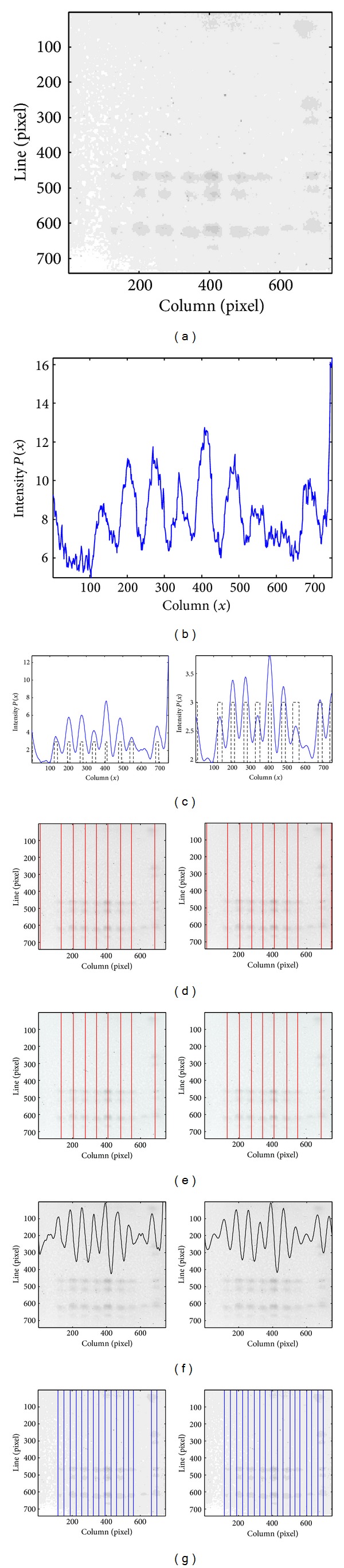
(a) ROI of the original TLC image. (b) Original intensity profile after data integration. (c)–(g) Intermediate and final results of [25] (left) and the herein proposed method (right).

For assessing the importance of the initial smoothing step, a comparison between the approach herein presented and the one described in [25] is shown in Figure 11. The main difference between these two methods is the initial smoothing step. The ROI of a DB1 image and its original intensity profile are shown in Figures 11(a) and 11(b), respectively. Figures 11(c) to 11(g) present intermediate results of both the methods proposed in [25] (left) and the herein described methodology (right). The profile smoothed using the SG filter still presents high frequency components that are enhanced in the profile derivative. As a consequence, the subtle lane included in the image is only segmented when the CWT-based smoothing is applied (Figure 11(g)).

**Figure 12:**
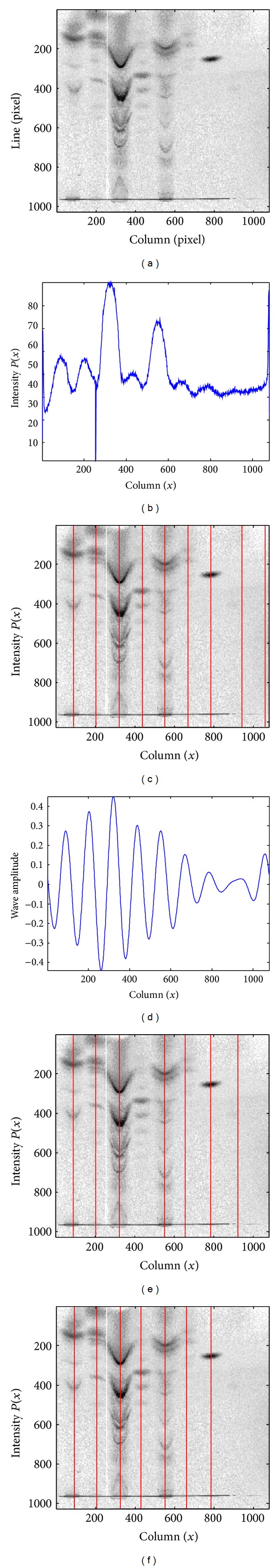
(a) ROI of the original TLC image. (b) Original intensity profile. (c) Detected lanes using the approach described in [18]. (d) Profile obtained using the approach described in [18]. (e) Detected lanes using the approach described in [19]. (f) Detected lanes using the proposed methodology.

In Figure 12, the proposed methodology is compared with the ones described in [18, 19]. In [19], the intensity information is projected onto the horizontal direction, and afterwards, a nonweighted moving average filter is iteratively applied to the profile until the number of potential lanes, which is estimated based on the number of local maxima, remains unchanged. Each lane is delimited by two consecutive maxima of the profile, while the points where the projection is minimal are associated with lane central positions. We have implemented the method in [18] following the description found in the literature and using as an input parameter the total number of occupied lanes in the chromatographic plate.

In order to compare the performance of the methods, the results of the proposed methodology and the ones obtained with the methods described in [18, 19] are presented in Figure 12.  Figure 12(a) shows the original TLC image (which includes two empty lanes), with the respective intensity profile depicted in Figure 12(b). The set of lanes detected when using the methodology described in [18] is shown in Figure 12(c). This set includes two false detections resulting from local maxima of the CWT-based smoothed intensity profile presented in Figure 12(d). The number of false lane detections was reduced to one when the method described in [19] was applied, but a true lane was missed (Figure 12(e)). All lanes were correctly detected by the methodology proposed in this paper, as illustrated by Figure 12(f).

### 3.4. Comparative Analysis of Methods' Performance

This section is devoted to the analysis of the performance of methods mentioned in the previous subsections. The four approaches were applied to all the images of the two datasets described in Section 3.1. The parameters for our method were set as detailed in Section 3.2. For the method in [19], the code was provided by the author.

Tables 1 and 2 show the results obtained after each of the three phases of the described methodology, when applied to DB1 and DB2, respectively.

In order to get a more reliable evaluation of the influence of the smoothing phase, we have compared the results from the methods in [18, 19] with those obtained after the first phase for both the proposed methodology and the one described in [25]. Thus, the refinement steps (the second and third phases of lane segmentation) are excluded from the results, as the improvements of these two last lane segmentation phases could give an unfair advantage to the methods that use them. These results are presented in Tables 3 and 4. 

The final results obtained with the four methodologies are resumed in Tables 5 (DB1) and 6 (DB2).

## 4. Discussion

A robust smoothing technique combined with the last two phases of the lane segmentation process achieved the best overall performance for both datasets. From the results in Tables 1 and 2, it is possible to conclude that each of the refinement steps addresses a different problem. In DB1, because most of the true lanes were detected during the first phase of the lane segmentation process, the improvement introduced by the lane recovery step is residual, but several false lanes were removed during the second phase. The images in DB2 contain a significant number of subtle lanes that, although not included in the initial set of lanes, were recovered during the third phase.

The methodology described in [18] relies on the selection of a specific scale of the CWT, followed by a detection of local extremes on the profile, reconstructed using the coefficients associated with that scale. This characteristic of the method makes it more effective when the lanes to be segmented are uniformly distributed all over the image, creating a periodicity that matches the reconstructed profile, thus justifying the good performance on the detection of true lanes even when they are very subtle. Nonetheless, this same characteristic is responsible for the high number of false detections when the images contain large empty regions corresponding to empty lanes, as happens in some images of DB1.

The main drawback of the method in [19] is the attenuation of the small peaks caused by the iterative nonweighted average filtering, making the detection of subtle lanes a very hard task. Indeed, the majority of the 27 missed lanes that result from the application of this method to DB1 are associated with lanes represented by very low intensity peaks in the profile. In DB2, the detection of lanes in image extremes is also a problem, since most are not represented by a significant maximum in the intensity profile after filtering. 

The methodology described in [25] achieved good results with the DB1 images through the elimination of false lanes and the recovery of subtle ones, in spite of the use of identical parameter values for all the images in the dataset. However, when the methodology was applied to DB2 images, a lower performance was obtained, in the numbers of both false detections and missed lanes (Table 4).

Finally, the importance of the new smoothing solution based on the CWT is clearly demonstrated by the results obtained after the first phase of the lane segmentation process (Table 5), which outperform all the values achieved by the other three methods.

## 5. Conclusions

We have described a new methodology for lane detection in chromatography images using an innovative technique based on the CWT for decomposing the original signal, followed by the reconstruction of a new smoothed profile, using a set of coefficients in a selected range of scales adapted to each image. This technique has proven to be able to deal with the noise present in the intensity profile, while preserving the main features that are required for the subsequent three phases of the lane segmentation process. Although the first one relies on the detection of profile maxima as proposed in other solutions, the novelty of the methodology presented in this paper is the inclusion of two refinement stages, using parameters adapted to image features, to overcome some of the limitations of other approaches. The proposed methodology is fully automatic and does not require the number of lanes as an input, neither a constant spacing between lanes, nor the absence of empty lanes. The smoothing solution based on the CWT also proved to perform better than those found in [19, 25], even without the improvement introduced by the refinement phases. 

Lane segmentation is the initial phase of the development of a screening tool for genetic disorders, and in particular Fabry disease. However, this is a crucial part of the system, as an erroneous detection of lanes will prevent its use. As future work, we intend to develop advanced methods for the analysis of lane patterns aiming at automating the identification of major disease's biomarkers.

## Figures and Tables

**Algorithm 1:**
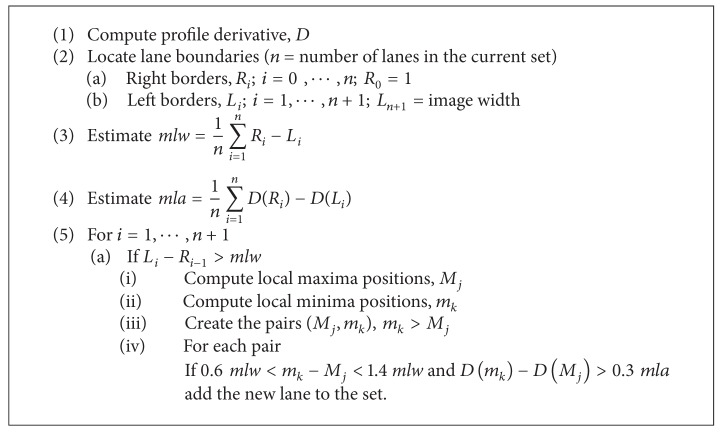
Subtle lane recovery.

**Table 1:** Results obtained for each phase of the proposed methodology, when applied to the images in DB1.

		True lanes detected	True lanes missed	False lanes detected	Recall (*R*)	Precision (*P*)	F_*β*_-measure (*β* = 1)
Real class	First phase	647	4	28	99.3%	95.8%	97.5%
	Second phase	647	4	12	99.3%	98.2%	98.7%
	Third phase	649	2	12	99.7%	98.2%	98.9%

**Table 2:** Results obtained for each phase of the proposed methodology, when applied to the images in DB2.

		True lanes detected	True lanes missed	False lanes detected	Recall (*R*)	Precision (*P*)	F_*β*_-measure (*β* = 1)
Real class	First phase	1350	72	46	94.9%	96.7%	95.8%
	Second phase	1348	74	37	94.7%	97.3%	96.0%
	Third phase	1395	27	38	98.1%	97.3%	97.7%

**Table 3:** Comparison of final results obtained using the methods [18] and [19] with the intermediate values after the first phase of both the proposed methodology and that described in [25] (DB1).

		True lanes detected	True lanes missed	False lanes detected	Recall (*R*)	Precision (*P*)	F_*β*_-measure (*β* = 1)
Real class	Akbari et al. [18]	480	171	140	73.7%	77.4%	75.5%
	Sousa et al. [19]	622	29	58	95.6%	91.4%	93.5%
	Moreira et al. [25]	644	7	31	98.9%	95.4%	97.1%
	Proposed method	647	4	28	99.3%	95.8%	97.5%

**Table 4:** Comparison of final results obtained using the methods [18] and [19] with the intermediate values after the first phase of both the proposed methodology and that described in [25] (DB2).

		True lanes detected	True lanes missed	False lanes detected	Recall (*R*)	Precision (*P*)	F_*β*_-measure (*β* = 1)
Real class	Akbari et al. [18]	1359	62	49	95.6%	96.5%	96.0%
	Sousa et al. [19]	1302	120	11	91.2%	99.2%	95.0%
	Moreira et al. [25]	1316	106	75	92.5%	94.6%	93.5%
	Proposed method	1350	72	46	94.9%	96.7%	95.8%

**Table 5:** Results obtained for the three methodologies on DB1.

		True lanes detected	True lanes missed	False lanes detected	Recall (*R*)	Precision (*P*)	F_*β*_-measure (*β* = 1)
Real class	Akbari et al. [18]	480	171	140	73.7%	77.4%	75.5%
	Sousa et al. [19]	622	29	58	95.6%	91.4%	93.5%
	Moreira et al. [25]	647	4	20	99.4%	97.0%	98.2%
	Proposed method	649	2	12	99.7%	98.2%	98.9%

**Table 6:** Results obtained for the three methodologies on DB2.

		True lanes detected	True lanes missed	False lanes detected	Recall (*R*)	Precision (*P*)	F_*β*_-measure (*β* = 1)
Real class	Akbari et al. [18]	1359	62	49	95.6%	96.5%	96.0%
	Sousa et al. [19]	1302	120	11	91.2%	99.2%	95.0%
	Moreira et al. [25]	1360	62	54	95.6%	96.2%	95.9%
	Proposed method	1395	27	38	98.1%	97.3%	97.7%

## References

[1] Fabry H (2002). Angiokeratoma corporis diffusum-Fabry disease: historical review from the original description to the introduction of enzyme replacement therapy. *Acta Paediatrica*.

[2] Zarate YA, Hopkin RJ (2008). Fabry’s disease. *The Lancet*.

[3] Eng CM, Germain DP, Banikazemi M (2006). Fabry disease: guidelines for the evaluation and management of multi-organ system involvement. *Genetics in Medicine*.

[4] Schiffmann R, Kopp JB, Austin HA (2001). Enzyme replacement therapy in fabry disease a randomized controlled trial. *Journal of the American Medical Association*.

[5] Miller JM (2005). *Chromatography: Concepts and Contrasts*.

[6] Fried B, Sherma J (2005). *Thin-Layer Chromatography, Part I, Chapter 1*.

[7] Sewell PA, Wilson ID, Poole C (2004). Liquid chromatography: theory of liquid chromatography. *Handbook of Methods and Instrumentation in Separation Science*.

[8] Sherma J, Sherma J, Fried B (2003). Basic TLC techniques, materials, and apparatusin. *Handbook of Thin-Layer Chromatography*.

[9] Pizzonia J (2001). Electrophoresis Gel Image processing and analysis using the KODAK 1D software. *BioTechniques*.

[10] Zerr T, Henikoff S (2005). Automated band mapping in electrophoretic gel images using background information. *Nucleic Acids Research*.

[11] Cooper ML, Maffitt DR, Parsons JD, Hillier L, States DJ (1996). Lane tracking software for four-color fluorescence-based electrophoretic gel images. *Genome Research*.

[12] Pavel AB, Vasile CI (2012). PyElph-a software tool for gel images analysis and phylogenetics. *BMC Bioinformatics*.

[13] Wong RTF, Flibotte S, Corbett R (2010). LaneRuler: automated lane tracking for dna electrophoresis gel images. *IEEE Transactions on Automation Science and Engineering*.

[14] Machado AMC, Campos MFM, Siqueira AM, De Carvalho OSF Iterative algorithm for segmenting lanes in gel electrophoresis images.

[15] Elder JK, Southern EM, Bishop MJ, Rawlings CJ (1987). Computer-aided analysis of one dimensional restriction fragment gels. *Nucleic Acid and Protein Sequence Analysis-A Practical Approach*.

[16] Bajla I, Holländer I, Fluch S, Burg K, Kollár M (2005). An alternative method for electrophoretic gel image analysis in the GelMaster software. *Computer Methods and Programs in Biomedicine*.

[17] Lin C, Ching Y, Yang Y (2007). Automatic method to compare the lanes in gel electrophoresis images. *IEEE Transactions on Information Technology in Biomedicine*.

[18] Akbari A, Albregtsen F, Jakobsen KS (2010). Automatic lane detection and separation in one dimensional gel images using continuous wavelet transform. *Analytical Methods*.

[19] Sousa AV, Aguiar R, Mendonça AM, Campilho AC, Campilho AC, Kamel MS (2004). Automatic lane and band detection in images of thin layer chromatography. *Image Analysis and Recognition, LNCS, 3121*.

[20] Maramis C, Delopoulos A Efficient quantitative information extraction from PCR-RFLP gel electrophoresis images.

[21] Maramis C, Karagiannis D, Delopoulos A, Broeck DV (2012). HPVTyper: a software application for automatic HPV typing via PCR-RFLP gel electrophoresis. *Human Papillomavirus and Related Diseases-from Bench to Bedside-Research Aspects*.

[22] Park SC, Na IS, Kim SH Lanes detection in PCR gel electrophoresis images.

[23] Sotaquirá M On the use of distance maps in the analysis of 1D DNA gel images.

[24] Barrantes P, Alvarado P Lane detection on gel electrophoresis images using active shape models.

[25] Moreira BM, Sousa AV, Mendonça AM, Campilho AC, Campilho AC, Kamel MS (2012). Automatic lane detection in chromatography images. *Image Analysis and Recognition, LNCS 7325*.

[26] Savitzky A, Golay MJE (1964). Smoothing and differentiation of data by simplified least squares procedures. *Analytical Chemistry*.

[27] Chau F, Liang Y, Gao J, Shao X (2004). *Chemometrics-from Basics to Wavelet Transform, Chapter 4*.

[28] Sousa AV, Sá-Miranda MC, Mendonça AM, Campilho AC, Campilho AC, Kamel MS (2011). Classification-based segmentation of the region of interest in chromatographic images. *Image Analysis and Recognition, LNCS 6754*.

[29] Sedaaghi MH, WU QH, Heijmans HJAM, Roerdink JBTM (1998). The power of morphological filters alone and when combined with linear filtering. *Mathematical Morphology and Its Applications to Image and Signal Processing*.

[30] Addison PS (2005). Wavelet transforms and the ECG: a review. *Physiological Measurement*.

[31] van den Berg JC (2004). *Wavelets in Physics, Chapter 10*.

[32] Young RK (1993). *Wavelet Theory and Its Applications, Chapter 1*.

[33] Mallat S (2009). *A Wavelet Tour of Signal Processing, Chapter 1*.

[34] Daubechies I (1990). Wavelet transform, time-frequency localization and signal analysis. *IEEE Transactions on Information Theory*.

[35] Grinsted A, Moore JC, Jevrejeva S (2004). Application of the cross wavelet transform and wavelet coherence to geophysical times series. *Nonlinear Processes in Geophysics*.

[36] Soille P (2004). *Morphological Image Analysis-Principles and Applications, Chapter 6*.

[37] Sousa AV, Mendonça A, Campilho A (2008). Chromatographic pattern classification. *IEEE Transactions on Biomedical Engineering*.

